# Can we safely continue ADHD medication in pregnancy ?

**DOI:** 10.1192/j.eurpsy.2026.10353

**Published:** 2026-07-31

**Authors:** E. Di Giacomo

**Affiliations:** 1 ASST Fatebenefratelli Sacco; 2 University Milano Bicocca, Milan, Italy

## Abstract

**Abstract:**

Attention-deficit/hyperactivity disorder (ADHD) is one of the most common neurobehavioral disorders, and it afflicts about 7% of young people. Its prevalence in adult women is 3.2% in the US. As a consequence, many young women might be pregnant while taking medication for ADHD, but data about safety have not yet been strictly examined. To mend such gap, a serch throug Electronic databases (PubMed,Embase,andPsycINFO) were conducted through December 31, 2023. It resulted in a meta analysis of ten studies involving 16621481 pregnant women, 30830 of them affected by ADHD. Congenital anomalies or miscarriages were not more frequent in offspring of mothers receiving treatment with methylphenidate or atomoxetine during pregnancy compared with unexposed offspring (OR, 1.14; 95% CI, 0.83-1.55; P = .41; I2 = 8% for congenital anomalies; OR, 1.15; 95% CI, 0.82-1.62, P = .67; I2 = 0% for miscarriages) or compared with the general population (OR, 1.19; 95% CI, 0.93-1.53; P = .16; I2 = 74% for congenital anomalies; OR, 0.98; 95% CI, 0.77-1.25; P = .40; I2 = 0% for miscarriage). In conclusion, evidence suggests the maintenance of methylphenidate or atomoxetine during pregnancy is safe, sincecongenital anomalies and miscarriages did not appear to significantly increase. Further studies are highlyrecommended to support and confirm these findings.

**Disclosure of Interest:**

None Declared

